# Hospital Needs and Hospital Appeals

**Published:** 1915-12-18

**Authors:** 


### HOSPITAL NEEDS AND HOSPITAL APPEALS.

We publish a letter on p. 265 from an able correspondent, who once again exhibits an absence of apprehension of the real basis on which propaganda for our voluntary hospitals in the present day must rest, if public support in increasing volume is to be maintained. We give some striking figures in the note attached to our correspondent's letter which we commend to those interested.

We have been told occasionally by prejudiced people that the appeals and accurate information we publish from time to time cannot produce money, because The Hospital is a technical paper which circulates mainly amongst interested people. This type of Solon continuously complains that his institution does not receive many legacies, and by this plea he effectively reveals the value as a legacy inspirer of The Hospital and of "Burdett's Hospitals and Charities." Another criticism is that generous donors are not guided in their distribution of charity by the efforts of The Hospital nor by the audited information and accurate facts which constitute its value. This is a favourite contention in connection with the Christmas Appeal Number, and there are a few institutions which do not even trouble to send us information of their needs, though they might be applied to for a little munition to enable us to fight their battle and help their funds.

It may be well, therefore, to dispose of these fairy tales which rest upon narrow views and limited information and experience. A fortnight before Christmas a generous donor, who systematically gives on principle to charity, came to us with the request that we would edit his list of gifts for Christmas and the New Year. On examining the lists we found the total sum to be distributed amounted to £35,000, and that he had given at the end of each year from £25,000 to £50,000 in this way to hospitals and charities for fifteen consecutive years. This is not a solitary experience, nor has it been confined to any particular period of the year. The fact is that in the present day business men distribute their gifts on a business basis. They are accustomed to have before them audited statements of account and an elaborate analysis made of items of expenditure and other matters when estimating the value of the investments offered to them from time to time. Is it surprising that they should have so acquired the habit of basing their charity upon accurate information resting on audited figures and incontrovertible facts. Every year since The Hospital was published in 1886 we have done our best at this season of the year to reveal the needs of the principal metropolitan hospitals.

